# Tuftelin 1 Facilitates Hepatocellular Carcinoma Progression through Regulation of Lipogenesis and Focal Adhesion Maturation

**DOI:** 10.1155/2022/1590717

**Published:** 2022-06-19

**Authors:** Lei Zhu, Kai X. Zhou, Ming Z. Ma, Lin L. Yao, Yan L. Zhang, Hui Li, Chang Du, Xiao M. Yang

**Affiliations:** ^1^State Key Laboratory of Oncogenes and Related Genes, Shanghai Cancer Institute, Renji Hospital, School of Medicine, Shanghai Jiao Tong University, Shanghai 200240, China; ^2^Department of Infectious Diseases, Shandong Provincial Hospital Affiliated to Shandong First Medical University, Jinan, 250021 Shandong, China

## Abstract

Hepatocellular carcinoma (HCC) is the most common type of primary liver malignancy with poor prognosis worldwide. Emerging evidences demonstrated critical roles of lipid de novo synthesis in HCC progression, yet its regulatory mechanisms are not fully understood. Herein, we found that tuftelin 1 (TUFT1), an acidic phosphorylated glycoprotein with secretory capacity, was significantly upregulated in HCC and had an excellent correlation with patient survival and malignancy features. Through database mining and experimental validation, we found that TUFT1 was associated with fatty acid metabolism and promoted lipid accumulation in HCC cells. Further, we found that TUFT1 can interact with CREB1, a transcription factor for hepatic lipid metabolism, and regulate its activity and the transcriptions of key enzymes for lipogenesis. TUFT1 promoted HCC cell proliferation significantly, which was partially reversed by treatment of an inhibitor of CREB1, KG-501. Moreover, TUFT1 promoted the capacity of HCC cell invasion in vitro, which was likely mediated by its association with zyxin, a zinc-binding phosphoprotein responsible for the formation of fully mature focal adhesions on extracellular matrix. We found that TUFT1 can interact with ZYX and inhibit its expression and recruitments to focal complexes in HCC cells. Collectively, our study uncovered new regulatory mechanisms of TUFT1-mediated lipogenesis, cell proliferation, and invasion.

## 1. Introduction

Hepatocellular carcinoma (HCC) is the most common type of primary liver cancer accounting for one of the leading causes of death globally [1]. Chronic hepatitis caused by hepatitis B or C virus infection is the most prominent risk for the development of HCC [2]. A considerable number of studies have been undertaken aimed at understanding the HCC progression, while the mechanisms that mediate the pathogenesis of HCC are not fully understood, which poses a challenge to global health [3].

The liver is the reigning organ that plays crucial functions in lipid homeostasis regulation through complex processes. As the main liver parenchymal cells, hepatocytes take command of metabolic functions, including fatty acid (FA) and triglyceride (TG) metabolism [4]. There are two major sources of FA: the hydrolysis of esterified FA in TG transported by gut-derived chylomicrons and de novo lipogenesis from excess glucose in hepatocytes [5]. FAs contribute to many important cellular events, including the synthesis of cellular membranes and intracellular signaling transduction. The aberrant metabolism of liver is closely related to liver disease such as inflammation, fibrosis, and cancer [6]. Carcinogenesis and adaptation to tumoral microenvironment are partially fuelled by metabolic alterations [7]. The high “starvation” of lipids in tumors is mainly fulfilled by de novo lipogenesis, and consequently, FA oxidation can be also increased in several tumor types [8].

Data emerging in last few years clearly suggested that lipid synthesis and desaturation are indispensable in HCC progression. For example, FA synthesis-related genes, including acetyl-CoA carboxylase (ACC), ATP-citrate lyase (ACLY), and fatty acid synthase (FASN), are all upregulated in HCC. FAs can be converted to monounsaturated fatty acids (MUFA), therefore providing sources for triacylglycerol (TAG) generation. MUFA synthesis is catalysed by stearoyl-CoA desaturase (SCD) which is often upregulated in human HCC [9]. Transcription factor SREBP-1c has been demonstrated to regulate the expression of this critical enzyme for FA synthesis in HCC patients [10]. Cyclic AMP-responsive element binding protein 1 (CREB1), another transcription factor that binds with cyclic AMP response element, controls the expression of a great number of genes involved in growth and stress signals [11]. CREB1 plays a crucial role in lipid synthesis both in the liver and adipose of nonruminants. In goat mammary epithelial cells, overexpression of CREB1 markedly upregulates SREBP1 and lipogenesis enzymes including FASN and promotes MUFA biosynthesis [12]. Blocking FA synthesis pathways has exhibited promising effects in tumor suppression, and several proteins in these pathways (e.g., SCD) had been identified as potential targets in cancer [13, 14]. Thus, further investigations of FA biosynthesis machinery in HCC would benefit for development of new treatment strategies.

Focal adhesions are macromolecular assemblies associated with plasma membrane and surrounding extracellular matrix (ECM). They also physically link to the actin cytoskeleton by recruiting a number of associated proteins, thus serving as a linkage between ECM and actin cytoskeleton. In this way, focal adhesions integrate external signals into intracellular signaling networks to organize actin cytoskeleton, reshape cell morphology, and modulate cell behaviors [15]. The capacities of cell migration are critically regulated by the dynamics of focal adhesions and the strength of cell-substrate adhesion [16, 17]. Compromised cell-substratum adhesion with short-lived turnover results in increased cell motility in contrast to mature focal adhesions [18]. Paxillin (PAX) and zyxin (ZYX) are scaffolding proteins participating in the maturation of nascent focal adhesions. ZYX recruitment to focal adhesion foci is a relative late event that contributes to the formation of fully mature centripetal focal adhesions [19]. Thus, the existence of ZYX in focal adhesions might indicate a strong adhesion of cell to the matrix and suggests a decreased cell motility. Nevertheless, the regulatory mechanism of recruitment of ZYX to focal adhesions is not fully revealed.

Human tuftelin gene (TUFT1) is located at 1q21.3 and encodes a conserved novel acidic protein containing 390 amino acids. The tuftelin gene was firstly cloned from a human genomic library in Lambda Fix using the bovine tuftelin cDNA probe [20, 21]. Most studies about TUFT1 focused on calcification and mineralization of teeth [22], while its role in cancers has gained attention in recent years. Liu et al. found that TUFT1 was involved in the development of triple negative breast cancer through regulating Rab5/Rac1 and Rac1/beta-catenin pathway [23–25]. In thyroid carcinoma, TUFT1 can regulate the Akt-mTOR/GSK3*β* pathway to promote cell proliferation and invasion [26]. Moreover, Kawasaki et al. found that TUFT1 could interact with RABGAP1 to activate mTORC1 signaling and regulate lysosomal positioning and vesicular trafficking in lung and breast cancer cell lines [27]. In pancreatic cancer, TUFT1 was upregulated and promoted epithelial-mesenchymal transition through regulating HIF1-Snail pathway [28]. TUFT1 also participates in the regulation of HCC progression through different signaling pathways [29, 30], whereas its interactome and functional mechanisms have not been fully unrevealed.

In this study, we demonstrated significant upregulation of TUFT1 expression in HCC and revealed its close association with unfavorable clinical features and poor prognosis of patients. Moreover, we found that TUFT1 could interact with transcription factor CREB1 and facilitate lipid de novo synthesis and HCC cell proliferation, with ZYX to promote motility of HCC cells.

## 2. Materials and Methods

### 2.1. Clinical Samples

We collected HCC samples from Renji Hospital, School of Medicine, Shanghai Jiao Tong University. These samples were informed by the World Health Organization Collaborating Center for Research in Human Production authorized by the Shanghai Municipal Government. 202 paired HCC and paracancerous liver tissues were constructed into tissue microarray (TMA1) and used to detect TUFT1 expression by immunohistochemical staining and analysis of patients' survival. To evaluate the correlation of TUFT1 with patients' clinicopathological features, TMA2 containing 55 cases of HCC was included in the immunohistochemical staining together with TMA1. To compare the TUFT1 mRNA and protein expression levels in HCC and corresponding noncancerous tissues, 14 freshly frozen HCC tissues were collected for extraction of RNA and total proteins, respectively.

### 2.2. Immunohistochemical Staining

Prepared slides were deparaffinized and rehydrated. After antigen repairing and endogenous peroxidase blocking with 0.3% hydrogen peroxide, slides were incubated with 10% BSA to prevent nonspecific binding, followed by incubation with primary antibodies overnight at 4°C and with HRP-conjugated secondary antibodies for 1 hr. Immunostaining was detected using DAB substrate (Thermo Scientific, USA) and counterstained with hematoxylin. Immunostaining was scored by two senior pathologists in a blinded manner, according to the ratio and intensity of the positive staining (score 1: <25%; score 2: 25-50%; score 3 : 50-70%; and score 4: >70%). All the sections were subdivided into two groups according to their scores: low expression (scores 1 and 2) and high expression (scores 3 and 4).

### 2.3. Cell Cultures

SMMC-7721, Huh7, MHCC-97H, and LM3 cells were obtained from the Shanghai Institute of Biochemistry and Cell Biology, Chinese Academy of Science (Shanghai, China), and cultured in Dulbecco's modified eagle's medium containing 10% FBS, 0.1 mg/ml streptomycin, and 100 units/ml penicillin at 37°C under 5% CO_2_.

### 2.4. Quantitative Real-Time PCR

Total RNA was extracted from human tissues or HCC cells using TRIzol reagent (Invitrogen, Carlsbad, CA, USA). Then, RNA was reverse transcribed by Prime Script RT-PCR (Takara, Japan) following the manufacturer's instruction. Real-time quantitative PCR were performed using SYBR Premix Ex Taq (Takara, Japan) on a 7500 real-time PCR system (Applied Biosystems). The cycling settings were as follows: denaturation at 95°C for 30 sec, followed by 40 cycles of 5 sec at 95°C and 31 sec at 60°C. The primers were listed in Table S1. The relative expression of genes was analyzed using 2^-△△Ct^ method using *β*-actin as the internal control.

### 2.5. Lentivirus Production and Cell Transduction

The full-length cDNA encoding human TUFT1-Flag was cloned into pCDH-CMV-MCS-EF1-Puro and shRNA constructs against TUFT1 were cloned into pLKO.1. Then, the lentiviral vectors were cotransfected with packaging vectors psPAX and pMD2.G into 293T cells using Jetprime reagent (Polyplus). 48 hours later, lentiviruses were harvested and virus titers were measured. HCC cells were infected with 1 × 10^6^ recombinant lentivirus-transducing units plus 6 *μ*g/ml polybrene (Sigma, Louis, MO, USA).

### 2.6. Cell Viability and Colony Formation Assays

For cell proliferation, HCC cells seeded in a 96-well plate at 2,000 cells per well were cultured in complete medium. Cell viability was detected at the indicated time points, with incubation of Cell Counting Kit-8 (CCK-8, WST-8, Dojindo, Japan) solution for 1 h at 37°C. Cell viabilities were determined by measuring the absorbance at 450 nm with a microplate reader.

For clonogenic assay, the cells were seeded into a 6-well plate at 1,000 cells per well. The cells were cultured for 14 days and then stained with crystal violet (Beyotime Institute of Biotechnology, Shanghai, China). The clones were photographed and counted for analysis.

### 2.7. Western Blotting

Cells lysates were prepared by RIPA buffer containing protease inhibitors. Denatured lysates were separated by 10% SDS-PAGE gel electrophoresis and transferred onto PVDF membranes (Millipore Corp, Billerica, MA, USA). After blocking with 5% nonfat milk, the membranes were incubated with primary antibodies overnight at 4°C. The following antibodies were used: anti-TUFT1 (1: 500 dilution, Santa Cruz), anti-phospho-CREB1 (Ser133, 1 : 1000, ImmunoWay), anti-ZYX (1 : 1000, Proteintech), and anti-*β*-actin (1 : 1000 dilution, Santa Cruz). After washing with TBS/Tween-20, the membranes were incubated with IRDye 680 anti-mouse or IRDye 800 anti-rabbit (LI-COR) secondary antibodies for 1 hr at room temperature. The bands were detected by Odyssey imaging system (LI-COR).

### 2.8. Subcutaneous Xenograft Model

Male nude (nu/nu) mice (SLAC, Shanghai, China) 6 weeks of age were injected subcutaneously in the lower back with a total of 1 × 10^6^ 97H shCtrl or shTUFT1 cells in 100 *μ*l Hanks buffered saline. Mice were sacrificed 4 weeks postinjection. Tumors were isolated and their weights were measured. Mice were housed and handled according to protocols approved by the East China Normal University Animal Care Commission.

### 2.9. In Vitro Invasion Assay

Matrigel mixed with DMEM media was added into the chambers of upper transwell. 6 × 10^4^ HCC cells suspended in DMEM were placing onto the Matrigel. After 48 hours, the cells invaded through the membrane pores were fixed by 4% formaldehyde solution, stained with crystal violet, and counted under microscopy (at least five fields per well). All assays were independently repeated three times.

### 2.10. Lipid Droplet Staining

For oil red O staining, HCC cells were firstly fixed in 4% paraformaldehyde solution for 15 min and washed with 60% isopropanol for 5 min at room temperature. Then, the cells were stained with ORO solution for 20 min and washed with distilled water. The nucleus was counterstained with hematoxylin. Intracellular lipid accumulation was examined using a light microscope.

For BODIPY 493/503 dye staining, HCC cells were seeded in a 12-well u-Chamber (ibidi, Germany). Then, the cells were fixed in 4% paraformaldehyde solution and immersed in BODIPY 493/503 solution for 30 min. The nucleus were labeled with 2 *μ*g/ml DAPI in PBS for 5 min. The images of droplets were acquired using confocal-scopy, Carl Zeiss.

### 2.11. Free Fatty Acid Analysis

The free fatty acid assay kit (Jiancheng, Nanjing, China) was used to detect the level of FFA according to the manufacture's instruction. 2 × 10^6^ cells were collected and homogenized in 200 *μ*l chloroform/Triton X-100. In the assay, fatty acids can be converted to coenzyme A and oxidized, leading to the formation of optical color which is captured by measuring the absorbance at 546 nm with a microplate reader. Palmitic acid is used to generate a standard curve. Relative concentration of FFA in the test samples was calculated and normalized. Independent experiments were done in triplicate.

### 2.12. Immunoprecipitation

Briefly, cultured cells were lysated with lysis buffer and the supernatant was collected. Magnetic beads (Bimake, China) were crosslinked with an anti-Flag antibody, CREB1, or species-matched control IgG according to the manufacturer's instructions. Lysates were centrifugated, and supernatant was incubated with antibody-conjugated magnetic beads for 1 hour at 4°C with gently rotation. After washing with PBS for several time, the bound proteins were eluted from the beads and analyzed by western blot.

### 2.13. Immunofluorescence

The cells were washed with PBS and fixed with 4% paraformaldehyde for 15 min at room temperature. Then, the cells were permeabilized with 0.05% Triton X-100 for 1 min and blocked with 10% BSA for 1 hr. The primary antibodies against ZYX (1 : 00, Proteintech), p-CREB1 (1 : 100, ImmunoWay), and paxillin (1 : 100, Abcam) were diluted with 1% BSA and incubated with the cells at 4°C overnight. Then, the cells were washed with PBS and incubated with Alexa Fluor-594 or -488 conjugated species-matched secondary antibodies. Finally, the cell nucleus were stained with DAPI, and the immunofluorescence images were captured and analyzed with a confocal microscopy.

### 2.14. Statistical Analyses

Statistical analyses were carried out using GraphPad Prism software. The relationship between TUFT1 expression and clinicopathological characteristics was analyzed by Pearson's chi-squared test. Student's *t*-test was used to compare the differences between two groups. Cumulative survival time was calculated by the Kaplan-Meier method and analyzed by the log-rank test. Values of *P* < 0.05 were considered statistically significant.

## 3. Results

### 3.1. TUFT1 Expression Is Increased in HCC and Correlated with Patients' Malignancy and Survival

**Figure 1:**
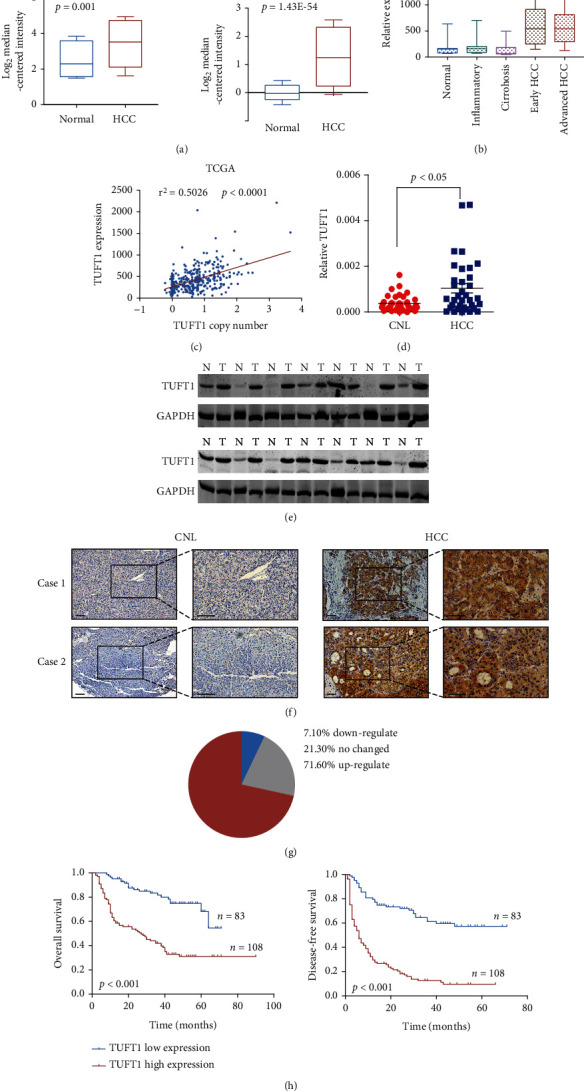
Expression of TUFT1 and its correlation with patients' survival in HCC. (a and b) Analysis of the expression of TUFT1 in HCC and corresponding normal tissue using three independent GEO datasets (GSE6764, GSE14520, and GSE54238). (c) Correlation analysis of TUFT1 expression and copy number alteration using TCGA database. (d and e) Relative mRNA (d) and protein (e) level of TUFT1 in HCC and corresponding normal livers. (f) Representative images of IHC staining of TUFT1 in the HCC tissue microarray (scale bar: 200 *μ*m). (g) Pie chart showing the percentage of cases with different TUFT1 expression profiles in comparison with CNL. (h) Kaplan-Meier survival analysis of patients with high or low TUFT1 expression, using TMA1 from Renji Hospital (Renji cohort).

By analyzing several HCC databases, we found that the mRNA level of TUFT1 is significantly increased in HCC tissues compared with normal counterparts (Figure 1(a)). We also analyzed GSE54238 which contains inflammatory, cirrhosis, early HCC, and late HCC samples and found that TUFT1 was increased both in early HCC and late HCCs but not in inflammatory or cirrhosis tissues (Figure 1(b)). Since TUFT1 gene is located at 1q21.3, the amplification region of HCCs [21], we analyzed its copy number and found that TUFT1 gene expression was significantly correlated with its copy number in TCGA HCC samples (Figure 1(c)), suggesting that TUFT1 upregulation could be attributed to gene amplification. qRT-PCR and western blot measurements confirmed its upregulation in HCC tissues compared with normal controls (Figures 1(d) and 1(e)). Further immunohistochemical staining of HCC tissue microarray containing 202 pairs of HCC and normal counterparts showed that TUFT1 was upregulated in 71.6% patients with dominant cytoplasmic expression in HCC cells (Figures 1(f) and 1(g)). Following Kaplan-Meier analysis demonstrated that TUFT1 expression was correlated with worse prognosis (Figure 1(h)). Following analysis of patient clinicopathological parameters showed that TUFT1 expression was closely associated with HCC tumor size, vascular invasion, and tumor differentiation (Table 1).

### 3.2. TUFT1 Promotes the In Vitro Proliferation and In Vivo Tumor Growth of HCC Cells

**Figure 2:**
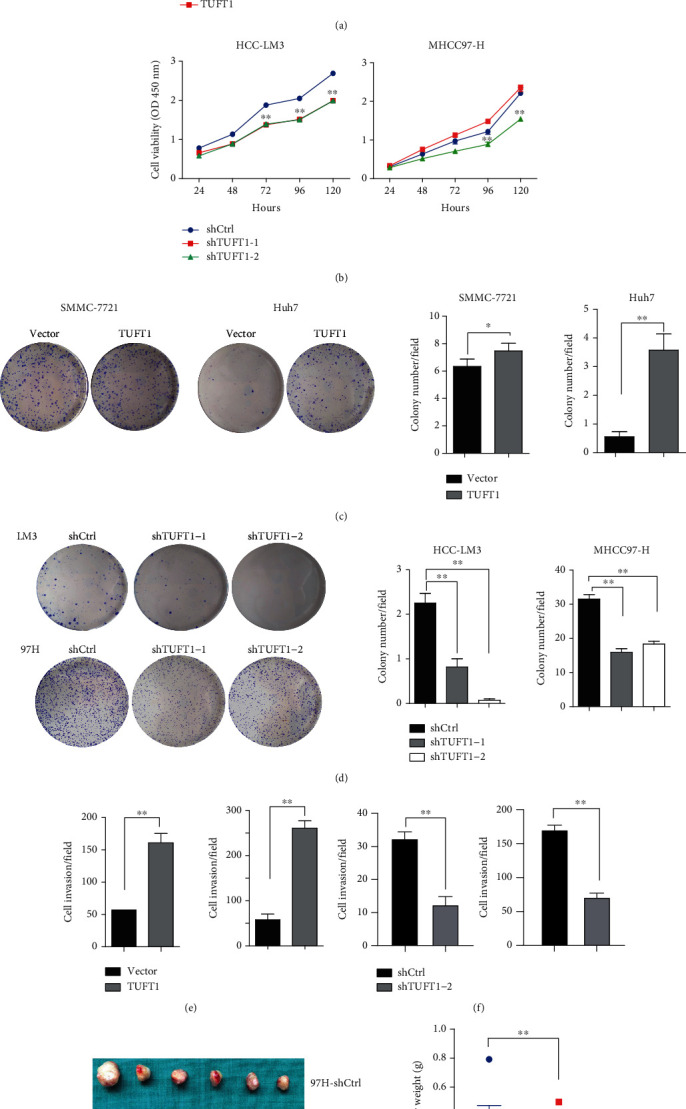
TUFT1 overexpression promotes while knockdown inhibits the oncogenic growth and invasion in HCC cells. (a and b) Cell proliferation analysis in TUFT1 overexpression or knockdown HCC cells. (c and d) Colony formation assay of TUFT1 overexpression or knockdown HCC cells. (e and f) Cell invasion analysis. (g) Tumor burden of control and TUFT1 knockdown cells induced subcutaneous xenograft model. Student's *t*-test: ^∗^*P* < 0.05 and ^∗∗^*P* < 0.01.

Next, we constructed pCDH-TUFT1-Flag plasmid which was transfected to 293T cells with package plasmids. The produced lentivirus was transduced to Huh7 and SMCC7721 cells for TUFT1 overexpression. We also constructed TUFT1 knockdown cell lines by transfecting PLKO.1-shTUFT1 lentivirus to MHCC97H and HCCLM3 cells (Supplementary Figure 1). In vitro cell viability and clone formation assay demonstrated that HCC cell proliferation was significantly facilitated by TUFT1 overexpression, while it was suppressed by TUFT1 knockdown (Figures 2(a)–2(d)). Cell invasion through Matrigel was also enhanced by TUFT1 overexpression and inhibited by TUFT1 knockdown (Figures 2(e) and 2(f)). Furthermore, subcutaneous xenograft model was established by injecting TUFT1 knockdown or control HCC cells, and the results showed that HCC tumor growth in vivo was also inhibited by TUFT1 knockdown (Figure 2(g)).

### 3.3. TUFT1 Expression Is Associated with Fatty Acid Metabolism in HCC Cells

**Figure 3:**
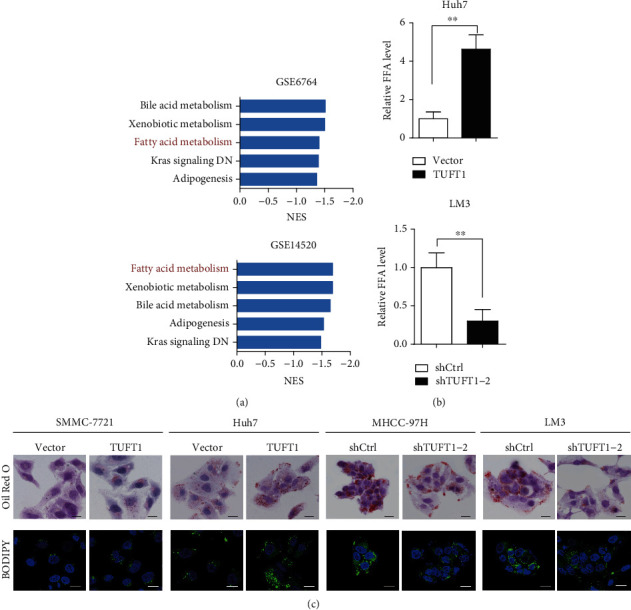
TUFT1 expression promoted lipid accumulation in HCC cells. (a) GSEA analysis showed that TUFT1 was involved in fatty acid metabolism. (b) Analysis of FFA level in TUFT1 overexpression or knockdown cells. (c) Lipid accumulation detected by oil red O and BODIPY 493/503 dye staining, in TUFT1 overexpression or knockdown cells. Scale bar: 10 *μ*m.

Next, to explore the underlying mechanisms mediated by TUFT1 in HCC, we performed GSEA enrichment analysis using GEO datasets and observed a significant enrichment of fatty acid metabolism in both GSE6764 and GSE14520 (Figure 3(a)). Dysregulated lipid level is a hallmark in cancer progression, and cancer cells can reprogram metabolic pathways to support rapid cell proliferation [29]. Elevated free fatty acid (FFA) provides substrates for energy production and generates lipid-signaling molecules to contribute to the malignancy of cancer cells [12]. To measure whether TUFT1 regulates fatty acid metabolism, we collected cell lysates of TUFT1-overexpressing and control Huh7 cells and measured for FFA levels. As shown in Figure 3(b), FFA levels were clearly increased in TUFT1-overexpressing Huh7 cells, and reversely, FFA level was decreased by TUFT1 knockdown in LM3 cells. Further, oil red O staining was performed in HCC cell lines with TUFT1 overexpression or knockdown. Consistently, the result showed that TUFT1 overexpression cells had obvious more and larger lipid droplets in cell cytoplasm than control cells, while those in TUFT1 knockdown cells were reduced (Figure 3(c)).

Next, we used BODIPY 493/503 dye staining to label cellular neutral lipid contents localized to lipid droplets. Consistent with oil red O staining, the results showed that TUFT1 overexpression was enhanced, while TUFT1 knockdown inhibited lipid deposition in HCC cell lines (Figure 3(c)).

### 3.4. TUFT1 Interacts with CREB1 and Promotes Its Phosphorylation and Nuclear Translocation

**Figure 4:**
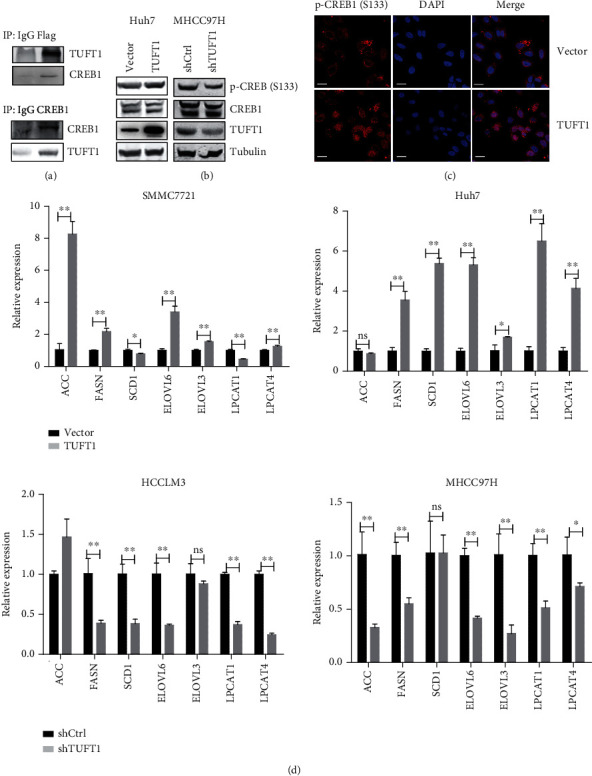
TUFT1 was associated with CREB1 and promoted its phosphorylation and nuclear translocation. (a) Co-IP of TUFT1-Flag with an anti-Flag antibody (upper panel), or with an anti-CREB1 antibody (down panel), followed by western blot analysis in Huh7 cells. (b) Western blot analysis of phospho- and total CREB1 in TUFT1-overexpressing or knockdown cells. (c) Immunofluorescence analysis of localization of phospho-CREB1 in TUFT1-overexpressing Huh7 cells. Scale bar: 20 *μ*m. (d) mRNA expression levels of lipid de novo synthesis enzymes semiquantified by qRT-PCR, using *β*-actin as an internal control. ns: no significance; ^∗∗^*P* < 0.01.

In cancer cells, including HCC cells, de novo fatty acid synthesis is the main source of cellular fatty acid and is commonly elevated [31]. We next set out to investigate whether TUFT1 contribute to de novo fatty acid synthesis. We found that hepatic lipid synthesis regulator CREB1 is associated with TUFT1 in the BioGRID protein-protein interaction database. Co-immunoprecipitation (co-IP) assay using an anti-Flag antibody in TUFT1-flag-overexpressing Huh7 cells was performed, and we found that CREB1 was captured by TUFT1-Flag (Figure 4(a)). Reversely, co-IP using an anti-CREB1 antibody showed that TUFT1 was also captured by CREB1 (Figure 4(a)). CREB1 is a transcription factor binding to the DNA cAMP response element and its activity dependent on its phosphorylation status. We found that CREB1 phosphorylation at Ser133 was enhanced by TUFT1 overexpression in Huh7 cells, while it was suppressed by TUFT1 knockdown in MHCC97H cells (Figure 4(b)). And we detected an obvious increase of nuclear translocation of CREB1 in TUFT1-overexpressing Huh7 cells (Figure 4(c)). These results demonstrate that TUFT1 has a regulatory role toward CREB1 transcription activity through associating with it and regulating its phosphorylation and nuclear localization.

CREB1 had been demonstrated to promote de novo fatty acid synthesis in different cell contents through transcriptional regulation of the enzymes responsible for de novo lipogenesis [32]. Using qRT-PCR analysis, we found the mRNA levels of a number of critical enzymes for de novo fatty acid synthesis were upregulated by TUFT1 overexpression and reduced by TUFT1 knockdown, including ACC, FASN, SCD1, ELOVL6, ELOVL3, LPCAT1, and LPCAT4 (Figure 4(d)). These results suggest that TUFT1 promotes FFA de novo synthesis through regulating transcriptions of lipogenesis enzymes, which may be dependent on the activity of CREB1.

### 3.5. Inhibition of CREB1 Partially Reverses TUFT1-Promoted Lipogenesis and HCC Growth

**Figure 5:**
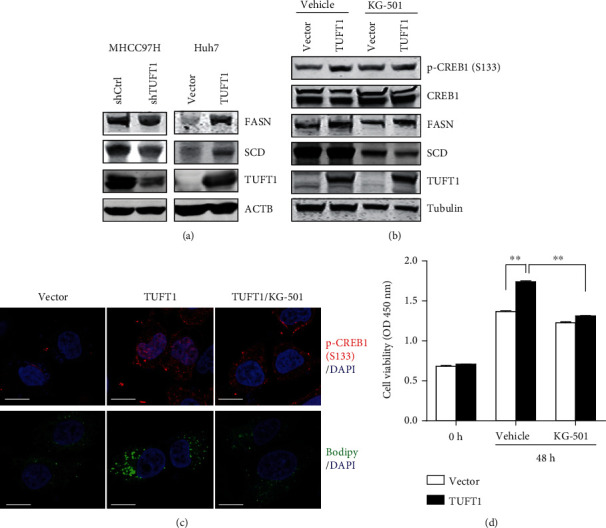
Inhibition of CREB1 reversed the promotion of cell proliferation by TUFT1 overexpression. (a) Western blot analysis of FASN and SCD in TUFT1-overexpressing or knockdown cells. (b) Western blot analysis of p-CREB1, FASN, and SCD in TUFT1-overexpressing Huh7 cells, treated with KG-501 or vehicle control. (c) Upper panel: immunofluorescence of p-CREB1 (Ser133) in vector, TUFT1 overexpression, and KG-501-treated TUFT1-overexpressing Huh7 cells; down panel: BODIPY 493/503 dye staining in these cells. Scale bar: 10 *μ*m. (d) Cell proliferation analysis of Huh7 cells, treated with KG-501 or vehicle control. ^∗∗^*P* < 0.01.

We further detected the expression of two key lipogenesis enzymes, FASN and SCD, by western blot and found that they were clearly increased by TUFT1 overexpression and reduced by TUFT1 knockdown (Figure 5(a)). To unveil the importance of CREB1 in mediating the function of TUFT1, we applied an inhibitor of CREB1, KG-501, in the experiments. We found that 10 *μ*M KG-501 had no impact on total CREB1 expression but partially reversed the upregulated phosphorylation of CREB1 in TUFT1-overexpressed cells. In addition, KG-501 also sharply reduced the expression of SCD (Figure 5(b)). Moreover, KG-501 treatment partially reversed TUFT1-induced CREB1 nuclear translocation and lipid accumulation in Huh7 cells (Figure 5(c)). Inhibition of CREB1 by KG-501 also significantly reversed the enhanced cell proliferation in TUFT1-overexpressing cells (Figure 5(d)). These results demonstrate that the promotional effect of TUFT1 on lipogenesis and HCC growth is partially dependent on CREB1 activity.

### 3.6. TUFT1 Suppresses ZYX Expression and Inhibits Its Recruitment to Focal Adhesion

**Figure 6:**
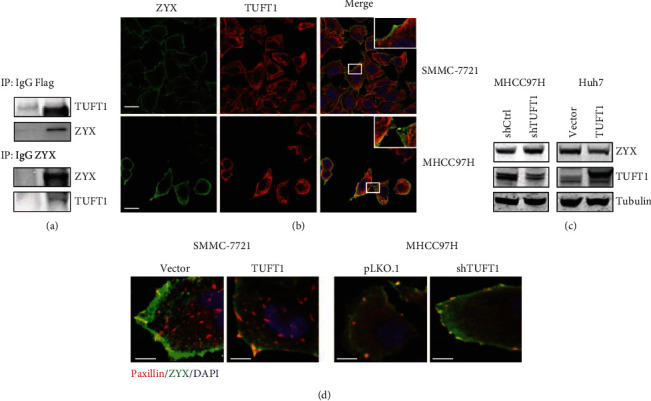
TUFT1 suppressed ZYX expression and inhibited its recruitment to focal adhesion. (a) Co-IP of TUFT1-Flag with an anti-Flag antibody (upper panel), or with an anti-ZYX antibody (down panel), followed by western blot analysis in Huh7 cells. (b) Co-immunofluorescence analysis of ZYX and TUFT1 in HCC cells, with colocalization at focal adhesions enlarged with a white rectangle; scale bar: 10 *μ*m. (c) Western blot analysis of ZYX in TUFT1-overexpressing or knockdown cells. (d) Co-immunofluorescence analysis of ZYX and paxillin in TUFT1-overexpressing or knockdown HCC cells. Colocalization in yellow at cell-substratum adhesion showed mature focal adhesions. Scale bar: 5 *μ*m.

Cell adhesion to the extracellular matrix (ECM) regulates a variety of cellular activities, including cell proliferation, migration, and death. The maturation of nascent focal adhesion is achieved by sequential recruitment of a number of scaffolding proteins including paxillin (PAX) and zyxin (ZYX). Recruitment of ZYX is a late event that is involved in the formation of fully mature centripetal focal adhesions. ZYX is another protein associated with TUFT1 analyzed from BioGRID. Using co-IP experiments, we found that ZYX can be captured by TUFT1 and vice versa (Figure 6(a)). Immunofluorescence analysis showed that ZYX was colocalized with TUFT1 at focal adhesions (Figure 6(b)). Western blot analysis demonstrated that ZYX expression was reduced by TUFT1 overexpression and enhanced by TUFT1 knockdown. Moreover, using co-immunofluorescence analysis, we found that ZYX recruitment in paxillin-positive focal adhesions was reduced in TUFT1-overexpressing cells, while it was increased in TUFT1 knockdown cells (Figure 6(c)). These results suggest that TUFT1 may impair focal adhesion maturation through inhibiting ZYX recruitment to focal adhesion, thus leading to increased capacity of HCC cell motility.

## 4. Discussion

Dysregulation of fatty acid (FA) metabolism is an emerging hallmark of malignant cells and compelling evidences indicated that it is involved in HCC development and progression [33, 34]. Obviously, continuous provision of energy and macromolecular building blocks is required for the rapid and uncontrolled proliferation of cancer cells. There are three source of FAs for HCC cells, including de novo FA synthesis, lipolytic pathway, and uptake from the external environment. Enhancement of *de novo* FA synthesis is a remarkable feature of HCC which involves significant increases in the expression of critical lipogenic enzymes, including FASN and SCD [35, 36]. In our present study, we found that TUFT1, a secretory protein, is capable of regulating the expression lipogenic enzymes. TUFT1 can associate with CREB1, a transcription factor of lipid metabolism, and increase its activity, thereby promoting FA synthesis in HCC progress. Besides, TUFT1 suppressed ZYX expression and inhibited its recruitment to focal adhesion, suggesting that TUFT1 could increase the capacity of HCC cell motility by impairing focal adhesion maturation.

Given the crucial contribution of accelerated FA synthesis to HCC carcinogenesis, interfering with lipogenesis is considered as potential therapeutic strategy for HCC. FASN is a notable target that has shown evident promotion in Akt-driven HCC, and blocking its activity exhibits great inhibition of HCC progression in mouse models [36]. Mounting investigations had led to the identification of a number of FASN inhibitors with antitumor activity preclinically, including GSK2194069, TVB-2640, C75, and orlistat [37, 38]. However, most of them were hindered from clinical trials due to unfavorable toxicities. On the other hand, ACLY inhibitors have shown effectiveness as cholesterol-lowering drugs in clinical trials of hypercholesterolemia [39, 40]. Currently, there is only a little advancement in the development of targeted therapeutics in cancers, and further investigations of the mechanisms of lipogenesis in HCC would be definitely helpful for developing new strategies for HCC treatment. TUFT1 is an acidic phosphorylated glycoprotein that is frequently overexpressed in human cancers. In this study, we found that TUFT1 high expression is closely related to malignant features and behaviors of HCC cells. TUFT1 promotes FA accumulation and the expression of several key lipogenic enzymes including FASN and SCD in HCC cells. Therefore, targeting TUFT1 might be a promising strategy to for HCC treatment.

Transcription factor CREB1 regulates the expression of numerous genes involved in growth factors and stress signals [41]. CREB1 is a phosphorylation-dependent transcription factor frequently upregulated in human cancers, including HCC. CREB1 phosphorylation can be activated by cAMP, calcium influx, or hormones through multiple pathways [41, 42]. CREB1 is involved in cellular processes through transcription regulation of target genes, including promoting the expression of protooncogene such as cyclin A [43, 44]. In colon cancer cells, phosphorylation of CREB1 was induced by norepinephrine to facilitate cell proliferation, migration, and invasion [45]. Besides, in prostate cancer cells, abiraterone acetate treatment can induce CREB1 phosphorylation, which in turn enhances CBP/p300 activity and lead to alterations in global gene expression and subsequently drug resistance [46]. Moreover, studies have demonstrated an indispensable role of CREB1 in lipid synthesis in liver and adipose in nonruminants. Overexpression of CREB1 also markedly upregulated SREBP1, FASN, and other key enzymes and promotes the synthesis of MUFA and accumulation of TG in mammary epithelial cells of goat [32]. CREB1 could also repress the expression of PPAR-gamma, a key regulator of lipogenic genes, to control hepatic lipid metabolism during fasting [47]. In yellow catfish, lipid deposition in the liver tissues and hepatocytes was increased by oxidized fish oils via the CREB1-Bcl2-Beclin1 pathway [48]. These studies revealed tight linkages between CREB1 and lipid metabolism in different contexts. In this study, we found that TUFT1 formed complexes with CREB1, increased its phosphorylation and nuclear translocation, and promoted expressions of lipogenic enzymes and lipid accumulation in HCC cells. Our results implied that TUFT1 might contribute to HCC proliferation through regulating CREB1-mediated lipogenesis. However, further investigations are needed for better understanding the regulatory mechanisms how TUFT1 regulates CREB1 activation and for further confirmation of the importance of CREB1 in mediating lipogenesis in HCC.

Regulation of focal adhesion proteins plays a key role in HCC progression by affecting cell motility and cell-substratum adhesion [49]. ZYX was commonly localized in mature centripetal focal adhesions, and incompetent to recruit ZYX would make the cell-substratum adhesion unstable and higher turnover, which would contribute to increased cancer cell motility and invasiveness [18]. In this study, we found that TUFT1 was a potential interactor of ZYX that suppressed its expression and recruitment to focal adhesions. These observations suggest that TUFT1 promotes HCC invasiveness through regulating ZYX-mediated cell-substratum adhesions. Nevertheless, the underlying mechanism how TUFT1 regulates ZYX is still unclear, which needs further investigations.

## 5. Conclusions

In conclusion, we demonstrated that increased TUFT1 was closely correlated with HCC patients' malignancy features and poor outcome. TUFT1 could interact with transcription factor CREB1 and promote its activity for lipid de novo synthesis and HCC cell proliferation. Moreover, TUFT1 interacts with ZYX and suppresses its recruitment to focal adhesions, thereby promoting the motility of HCC cells. Our study provides new insights into the mechanisms of TUFT1-mediated malignancy progression of HCC.

## Figures and Tables

**Table 1:** Correlations of TUFT1 with clinical pathological features of HCC patients.

Variable	TUFT1 (*n* = 257)		
	High	Low	*P* value
Age			
≤50 years	31	92	0.059
>50 years	49	85	
Gender			
Female	10	24	0.064
Male	70	153	
Liver cirrhosis			
Yes	61	143	0.039
No	19	34	
Tumor differentiation			
I	5	8	0.037
II	35	72	
III	40	96	
Vascular invasion			
Yes	50	53	0.0027
No	30	124	
Tumor size			
≤ 5 cm	57	50	0.0017
> 5 cm	35	115	
TNM stage			
I	49	110	0.0559
II	13	18	
III	18	49	

## Data Availability

The data used to support the findings of this study are available from the corresponding author upon request.

## Supplementary Materials

Supplementary Materials “See Figures S1 and Table S1 in the Supplementary Material for the expression analysis of TUFT1 and primer sequence, respectively.”
