# Supplementary material for: Tuftelin 1 Facilitates Hepatocellular Carcinoma Progression through Regulation of Lipogenesis and Focal Adhesion Maturation
Source: J Immunol Res. 2022 Jun 19;2022:1590717. doi: 10.1155/2022/1590717 (PMC9234046; doi:10.1155/2022/1590717)
Supplement: Supplementary Materials — “See Figures S1 and Table S1 in the Supplementary Material for the expression analysis of TUFT1 and primer sequence, respectively.” [file 1590717.f1.docx]

**Supplementary data**


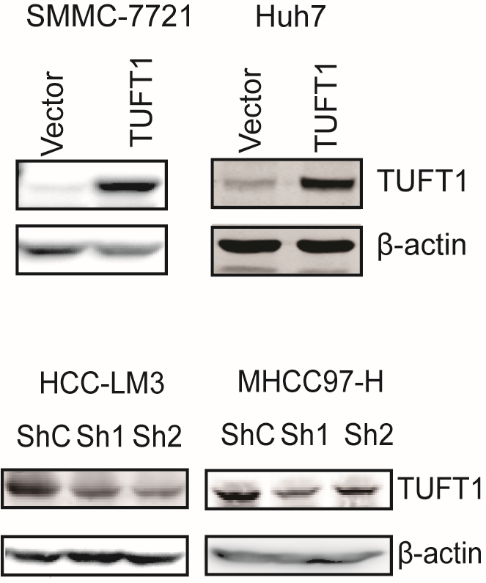


Supplementary Figure 1. Validation of TUFT1 overexpression or knockdown by western blot analysis in HCC cells.

Table S1. Sequences of qPCR primers

| Gene | Sequences (5’→3’) |
| --- | --- |
| LPCAT4 | F: GGCCTTTATCGTCCTCTTTCTC |
|  | R: CATCCTGTAATTGGCTCCTGAAG |
| LPCAT1 | F: TTACCTTCAAACCTGGTGCATT |
|  | R: CGTGAGCCACAGGATTTCC |
| ACC | F: GCCCTTCTGAGTCGCTTAATATG |
|  | R: TGACATCACCCCTAGAGTCCT |
| ELOVL6 | F: AACGAGCAAAGTTTGAACTGAGG |
|  | R: TCGAAGAGCACCGAATATACTGA |
| ELOVL3 | F: GTATTGGGCAACCTCATTCCC |
|  | R: CCTTGCGTTCCTTCATGTAGT |
| FASN | F: AAGGACCTGTCTAGGTTTGATGC |
|  | R: TGGCTTCATAGGTGACTTCCA |
| SCD1 | F: TTCCTACCTGCAAGTTCTACACC |
| TUFT1 | R: CCGAGCTTTGTAAGAGCGGT  F: GAAGATGAACGGGACGCGTAACTGGTG  R: CAAGTTCATCTCCTGTCAGTTCAC |
| β-actin | F: AGTTGCGTTACACCCTTTCTTG |
|  | R: CACCTTCACCGTTCCAGTTTT |
